# Exploring veterinarians’ perceptions of decision-making with clients in the context of providing access to veterinary care

**DOI:** 10.1371/journal.pone.0342564

**Published:** 2026-02-12

**Authors:** Catherine N.H. Groves, Jason B. Coe, Cathy Bauman, Lauren E. Grant

**Affiliations:** Department of Population Medicine, Ontario Veterinary College, Guelph, Ontario, Canada; Jaramogi Oginga Odinga University of Science and Technology, KENYA

## Abstract

Veterinarian-client decision-making and its role in improving accessibility of veterinary care for all pets is an emerging topic in the veterinary field. The objective of the present study was to understand veterinarians’ perceptions and experiences engaging in decision-making with clients in the context of providing access to veterinary care. Semi-structured interviews were conducted with companion-animal veterinarians (n = 15), randomly sampled from across Ontario. Inductive thematic analysis was conducted on verbatim transcripts. Most participants worked strictly as a small-animal practitioner at a multi-vet practice, were women, and employed full-time. Four inductive themes relating to the primary study objective were identified: 1. discussing options helps address barriers to veterinary care, 2. veterinarians’ descriptions of presenting options differed, 3. veterinarians and veterinary-team members face challenges in presenting options to clients, and 4. a recognized shift is happening away from a singular “Gold Standard” option. All participants discussed the importance of options and shared that discussing options assists in addressing access barriers to veterinary care. Participants differed in their descriptions of what presenting options to clients meant. Descriptions included A) presenting all available options alongside risks, benefits, and other details; B) making a recommendation and discussing other options, if needed; or C) contextualizing options based on history gathered prior to discussion of options with the client. Participant-identified challenges regarding discussion of options included time constraints, client non-cooperation, and veterinarian bias. All participants referenced the term “Gold Standard”, with many acknowledging a shift away from this practice toward offering a range of options in support of providing access to veterinary care. Presenting options and working with clients to contextualize care offers a potential opportunity to increase access to veterinary care.

## Introduction

Many pet owners face barriers to accessing veterinary care (AVC) [1]. A recent study estimated that 1.62 million pet-owning households (i.e., with a dog or cat) in Canada face barriers to receiving preventive care, 1.08 million face barriers to problem (i.e., sick) care, and 719,000 to emergency or urgent care [1]. Various barriers to AVC have been recognized, including the financial costs and affordability of care, geographic access and physical challenges to patients receiving care in a timely manner (e.g., geographical location, transportation of the animal, appointment availability), barriers specific to the animal (e.g., behavioral concerns, species-specific welfare needs), and communication or knowledge-based barriers (e.g., cultural/linguistic barriers, lack of client education) [1–4]. Barriers within most of these categories (e.g., affordability, knowledge-based barriers) can often be addressed or influenced by a veterinarian’s and client’s approach to decision-making. A number of terms have been used to introduce approaches to accessing veterinary care, including Incremental Care, Contextualized Care, and Spectrum of Care, all of which have been described to utilize shared decision-making (SDM), or decision-making in general, to facilitate the creation of feasible, accessible care plans [5–8].

Veterinarian-client decision-making has been identified as an important area of study to address ongoing challenges of ensuring access to veterinary care [9]. Healthcare-provider approaches to decision-making have been described using 3 main models, from a paternalistic approach (i.e., the practitioner makes the decision, the client is notified), to an informed or consumeristic approach (i.e., client-led, with the practitioner providing technical and scientific knowledge for the client to consider) [10]. In-between lies a third model, the relationship-centered or SDM model (i.e., practitioner and client share information and collaborate to agree upon a decision) [10]. The exchange of information occurring through SDM conversations between veterinary professionals and clients has been posited to achieve decisions that are contextualized to patient and client considerations, improving client ability and commitment to adhere to a care plan and thereby improving care outcomes [11]. In addition, with information on client and/or patient access barriers brought forth through a shared decision-making approach, the opportunity for the veterinary professional and client to identify an accessible alternative for care becomes possible. Given the potential intersectionality of many potential access-to-care barriers (e.g., a diabetic cat with a needle-hesitant owner who is also balancing caring for an ill family member – impacting available client finances and time), veterinarian-client decision-making is likely to play a complex, yet pivotal role in supporting individual AVC solutions. Therefore, as recently identified [9], veterinarian-client decision-making is an important aspect of AVC to be further explored.

With the potential for veterinarian-client decision-making to improve AVC for clients and their animals, veterinarians’ perspectives on the topic are needed to inform an understanding of the role decision-making has in AVC and to guide future research and education on AVC within veterinary medicine. The objective of this study was to understand a sample of veterinarians’ perceptions and experiences engaging in decision-making with clients in the context of providing access to veterinary care.

## Methods

Fifteen online, semi-structured interviews with companion-animal veterinarians were conducted in 2 rounds, between May 18^th^ and August 1^st^ of 2023 and May 15^th^ and July 25^th^ of 2024. Study protocol was reviewed and approved by the University of Guelph Research Ethics Board (REB#22-11-031). The consolidated criteria for reporting qualitative research (COREQ) guidelines were followed [12].

### Study participants

Recruitment involved the use of a random-number generator (https://www.random.org/) to aid in a selection of a random sample of veterinarians whose contact information was publicly available through the College of Veterinarians of Ontario’s (CVO) website (https://www.cvo.org). At the time of initial recruitment, 5,292 companion-animal veterinarians with active licenses were alphabetically listed on the CVO website. The random-number generator was then used to select a number between one and the total number of veterinarians listed. As a result, every 48^th^ veterinarian on the alphabetically sorted list was contacted until recruitment was complete. Eligible participants met the following inclusion criteria: English-speaking, currently identified to be practicing companion-animal veterinary medicine, and had an active license in good standing with the CVO. Potential participants were contacted to participate via a phone call. A follow-up email was sent if they agreed to receive further information. Participating veterinarians were provided with a CAD $50 Amazon gift card as an incentive.

A link within the follow-up email directed participants to a Microsoft Booking (online, Microsoft Corporation) page to schedule a 1-hour interview at their convenience. Following a participant booking an interview, informed, written consent was obtained and participants were asked to complete a questionnaire, both through Qualtrics (Provo, Utah, USA). The questionnaire (available in S1 File) gathered relevant demographic information including gender (man, non-binary, woman, prefer to self-describe [open text], prefer not to say), current employment status (full-time [40 or more hours per week], part-time [up to 39 hours per week]), role at practice (owner, associate, locum, prefer to self-describe [open text]), whether they worked strictly as a companion animal practitioner (yes, no), average duration of scheduled appointments (in minutes), average cost of examination (open text in CAD$), and received communications training (yes, no). If respondents indicated they had received veterinary communications training, they were asked to describe said training (open text). Year of graduation from veterinary college (open text) was used to determine number of years the participant had worked in veterinary practice at the time of their interview.

### Interview structure

All semi-structured interviews followed the same prepared and standardized interview guide (Table 1) developed by two members of the research team [CNHG, JBC], involving open-ended questions and follow-up probes. Terms used in association with access to care, specifically Spectrum of Care (SpoC), as well as Incremental Care (IC) and Contextualized Care (CC), were introduced at the end of the interview and participants were prompted to discuss their thoughts on these terms including the definition of SpoC published by the AAVMC (Table 1). Three pilot interviews were conducted to assess the interview guide and incorporate feedback on how it could be improved prior to formal data collection. Next, six study interviews were conducted for an initial round of interviews, followed by a second round of nine interviews including additional probes designed to further explore specific ideas raised during the initial set of interviews.

**Table 1:** Interview guide questions for veterinarian interviews.

Tell me about, what you think contributes to positive interactions with clients for you. What are challenges you encounter when interacting with clients? Walk me through how you approach decisions with clients. What are your thoughts on the term ‘the gold-standard’? What are your thoughts on situations where clients might “do nothing” or generally not pursue the care you are recommending? How do you present value of your veterinary care to clients? So, an area that has gotten a lot of attention in the last little while is the idea of Incremental care, also known as a Spectrum of Care or Contextualized Care. What do those terms mean to you? I would like to share with you the definition of Spectrum of Care given by the AAVMC (American Association of Veterinary Medical Colleges). They acknowledge the definition of the Spectrum of Care to be a “*wide spectrum of diagnostic and treatment options [that veterinarians] can provide for their patients” (p. 1386, Stull et al., 2018). Practicing across the spectrum entails “providing a continuum of acceptable care that considers available evidence-based medicine while remaining responsive to client expectations and financial limitations” (p. 464, Fingland et al., 2021)*” [13–15]. This continuum includes monitoring or doing nothing, as well as euthanasia. (Provide term in ‘chat’ feature). So, what are your thoughts on this definition?

Interviews were conducted by the principal author (CHNG) via Teams (version 16.0, Microsoft Corporation) and were audio-video recorded. Member checking [16] involved the interviewer summarizing and reviewing the main points provided by participants at the end of each interview, to confirm the interviewer’s interpretation of the gathered information with the participant, to support the trustworthiness of the interviewer’s interpretation. CNHG initially transcribed the recordings to produce verbatim transcripts within Word (version 16.8, Microsoft Corporation). Researcher reflexivity was used to support internal validity [16], with CNHG keeping a reflexive journal throughout data collection, the interviews, and analysis, and being intentionally curious throughout.

### Thematic analysis

Verbatim transcripts were subsequently reviewed again along with the audio-video recordings to ensure accuracy of transcribed data, as well as to continue to familiarize the lead author (CNHG) with the data. Using NVivo (Version 12; QRS International), CNHG engaged with the transcripts, following the process of reflexive thematic analysis [17]. This process was chosen due to its ability to identify patterns of meaning across data, as well as its theoretical flexibility aligning with the use of a constructivist paradigm [17]. An iterative, inductive approach was used to develop initial codes and subsequently derive subthemes, and themes from the data. Thematic analysis was also used to independently analyze participant responses to the definition of Spectrum of Care (SpoC). Transcripts were initially coded by order of participant and then by question to initiate different ways of engaging with the data [16]. Peer debriefing was also used to support the credibility of interpretations [16], through review of selected transcript segments and discussion of codes between CNHG and JBC. No new codes emerged by the time of the tenth interview, indicating data saturation had been reached [18].

## Results

### Participants

A total of 46 veterinarians were randomly contacted, all responded, and 15 consented to participate in an interview (32.6%, 15/46). Interview duration averaged 63 minutes and ranged from 37 minutes to 107 minutes. Most participants worked strictly as a small-animal practitioner at a multi-vet practice, were women, and were employed full-time. Additional demographic details are provided (Table 2).

**Table 2:** Demographic for the 15 participating veterinarians.

		n (%)	Mean, Min-Max, Median, SD
Gender	Woman	12 (80.0)	
	Man	3 (20.0)	
Employment Status	Full-time (40 or more hours per week)	11 (73.3)	
	Part-time (up to 39 hours per week)	4 (26.7)	
Role at practice	Owner	7 (46.7)	
	Associate	6 (40.0)	
	Locum	1 (6.7)	
	Other (Medical Director)	1 (6.7)	
Practitioner type	Small animal	14 (93.3)	
	Mixed animal (25–50% of time is spent practicing small animal)	1 (6.7)	
Average Appointment Duration (in minutes)			32, 30-45, 30, 5.28
Average Consultation Fee (in CAD)			127.05, 85.25-250, 119, 42.46
Communications Training	Yes	8 (53.3)	
	No	7 (46.7)	
Years in practice			22, 2-55, 20, 15.95

### Thematic analysis

Thematic analysis of the veterinarian interviews revealed 4 overarching inductive themes: 1) discussing options helps address barriers to veterinary care, 2) veterinarians’ description of presenting options differed, 3) veterinarians and team members face challenges to presenting options to clients, and 4) a recognized shift is happening away from a singular “Gold Standard” option. As well, the additional question exploring veterinarians’ thoughts on the term Spectrum of Care was analyzed independently.

#### 1. Theme: Discussing options helps address barriers to veterinary care.

All participants shared that discussing options with clients assisted in addressing multiple barriers to care. For example, one participant shared, *“normally, they’ll* [the client] *tell you* [about their considerations] *once you go through the options” (P13).* When asked their thoughts about situations in which clients do not pursue recommended care, participating veterinarians shared how they have used the discussion of options to reach an acceptable quality of care despite client barriers and considerations. These barriers included cost of care, client and patient circumstances, client understanding of the importance of care, and challenges specific to the patient. Participants described utilizing discussion around options for care, when faced with barriers, by offering options that were diverse financially, accounted for the client’s situation and perspective, or addressed the patient’s behaviour or health. Each of these barriers, including participants’ description of how discussing options addressed the barrier, are described below.

**1.1 Subtheme: Options help address** *“cost of veterinary care” (P14)* **by being financially diverse:** Offering acceptable options that cover a range of costs was described by veterinarians as an approach that supported them in helping clients overcome financial barriers. A third of participants shared how discussing options is beneficial in all financial situations and not only with *“cost-conscious” (P4)* clients. Participants in every interview described instances where client finances restricted the medical options that could be pursued, or where a client’s budget constraints reduced available options or required adjustments of options. Participants provided examples such as prescribing a medication without bloodwork and prioritizing a single diagnostic over multiple. Over half of participants expressed that they believed current inflation was hindering clients’ ability to afford care for their pets and specifically shared the benefits of discussing options to address this current influence (i.e., inflation) on clients’ financial circumstances. Furthermore, a few participants noted that as care has advanced, it has also become more expensive; as such, participants recognized the role options play in addressing the availability of this rapid advancement. As one participant summarized, *“by offering multiple options I can cater to the people who want* [and can afford] *the best of the best and then the people who maybe don’t even have a house or something like that. So, it doesn’t seem elitist…veterinary care these days I feel like is becoming a ‘rich man’s game’” (P6).* Addressing when clients shared notable financial barriers, participants also described attempting to assist by presenting available financing options and provided examples such as payment plans and charities that provide financial assistance for veterinary care.

**1.2 Subtheme: Options help address the** *“swirl” (P8)* **around clients by accounting for context:** Participants described options as helping them and their clients sort through patient and client circumstances that can act as a barrier to patients gaining care; as one participant described, *“a lot of the time people are coming in with a swirl, like their whole everything that’s gone on in the last few days or hours or decades is all contributing to…what they need” (P8).* This participant listed examples: *“People are like ‘I’m moving tomorrow’ or ‘I’m going away’ or ‘I can’t handle this because my mom just died, and I’ve got this funeral’” (P8).* Almost half of participating veterinarians shared examples where factors limited what was feasible for a client in terms of options. Examples of limiting factors described by participants included logistical considerations such as clients being unable to make the trip to the clinic, client work schedules creating time constraints, and clients balancing other priorities impacting their ability to attend to their animal’s care. By knowing these factors, participants described how, in discussion with clients around options, they could discuss potential solutions, including utilizing clinic resources (e.g., participants mentioned keeping the pet in-hospital for monitoring) or engaging the client in identifying other resources (e.g., friends or family members). One participant articulated these examples: *“We talk about other options. So, maybe there’s somebody in the neighborhood who can come over and administer those* [medications]. *Is it possible for us to do it in the hospital?” (P2)* A handful of participants described the emotional connections clients have with their pets and how this influences clients’ interpretation of the impact of care, which can influence decision-making. Participants described clients as balancing the client–patient bond with a patient’s care, with financial considerations and other personal, external barriers. Participants provided moving and caring for a sick parent or child as examples of these external barriers. The client–patient bond was portrayed as motivating both clients and veterinarians to overcome barriers to the provision of care.

Nearly all participants shared that there were few instances, if any, when they could not adjust or find an option appropriate for a context, as long as clients were transparent in providing information regarding relevant barriers. However, one participant opposed this view, commenting on their perception of a veterinarian’s role in finding solutions that fit client-related barriers: *“I don’t think* [the] *veterinarian’s concern should be the…ability of the owner to deal with the problem” (P9).* Otherwise, participants expressed benefiting from drawing out client preferences, values, and barriers, where most participants described how this discussion influenced which alternatives they explored in detail. For those that described adapting their presentation of options based on history gathered prior to discussing options, this remained true where information gathered beforehand influenced their presentation of multiple options. A handful of participants expanded on this description to include the importance of maintaining awareness that client expectations, abilities, and situations may change. Almost half of participants described appreciation of client transparency in sharing their perspectives and barriers for consideration. A participant summarized this view, stating *“if you’re honest with me up front and we can just incorporate it* [information shared], *and then we’ll have better success” (P5).*

**1.3 Subtheme: Discussing the outcome of each option allows veterinarians to manage client preferences**: Over a third of participants described, that discussing options allowed them to adapt the options to their clients’ concerns and assist clients in seeing that providing certain care (i.e., in the form of one of the presented options) is important to the health of their animal. Most participants tied communicating the difference in value of care between options to making clients aware of the realistic outcome for each option, and used examples such as a diagnostic test may not reveal as much compared to another, or techniques for weight loss require more time. With this link delineated between the value of care and realistic expectations specific to the outcome of each option, participants described that communicating why a care recommendation is important and the risks of alternatives, including the option of not pursuing care, assists them in communicating the value of care, thereby assuaging client hesitations. One participant summarized that they communicate, *“this is what you* [the client] *should expect and this is what the improvement is going to be for your animal and stuff…So, when I’m presenting options, say you have a cruciate ligament* [tear]… [the veterinarian will] *give them the option of surgery and* [another option]. *And* [when the client asks about non-surgical options] *you* [the veterinarian] *will say, ‘well, he’ll never walk or be able to run’” (P3).* A few participants mentioned clients may be uncomfortable with treatment due to invasiveness for the patient, with participants giving the example of client discomfort regarding their pet undergoing surgical procedures. Exploring options was described as assisting in working with these client concerns, as reflected in one participant’s example: *“I find one thing that does happen a lot is they* [the client] *assume surgery is… super risky, right? But ‘do nothing’ is also super risky” (P12).* By exploring client preferences and providing appropriate information, participants aimed to aid clients in understanding why care options are important for their animal to inform client decisions. As one participant summarized, *“We have to be candid and direct about what’s possible…I often don’t know what their expectations are until they’ve talked to me” (P15)*.

**1.4 Subtheme: Options allow care to be individualized to the animal**: Over half of participants shared that they discuss animal-specific barriers with clients to contextualize options to the patient’s situation, such the patient’s age, behaviour, comorbidities, and work around those barriers by including options that circumvent or minimize the barriers posed by the patient. Participants provided examples of animal-specific barriers, such as an aggressive pet or animal that would be uncooperative regarding a specific mode of treatment administration or cases where a patient’s current health status puts them at risk for a specific option. One participant shared the following example: *“If you can’t put eye drops in this dog because he tries to bite you, well then, we’re not doing the best course of treatment for his dry eye, because you physically can’t do it” (P1).* As long as participants were aware of the pet’s preferences or related barriers, they indicated that they were able to find acceptable alternative options.

#### 2. Theme: Veterinarians’ description of presenting options varied.

Offering options was discussed in-depth in all interviews, arising organically in conversation around decision-making, prior to probing on the topic further. Participants described that the main benefits of discussing options include A) providing information for informed-client consent, B) offering the opportunity for clients to disclose barriers and other considerations, and C) alleviating suffering and providing care to patients, regardless of client circumstance. As one veterinarian summarized, capturing the view of almost all participants, *“If you’re not willing to present options, it’s almost like you’re not willing to take their* [the client’s] *situation into account to help their pet” (P11)*.

Most participants initially described presenting all available options, including their respective risks and benefits, as well as other details. After the discussion of options arose, participants were probed further where a divergence in their described approach to presenting options was identified. Some participants shared multiple approaches and highlighted situations where they employed different approaches to presenting options; as such, there was participant overlap in the different approaches described. Almost two-thirds of participants described always mentioning to the client, briefly or in detail, that alternatives are available; whereas approximately a third shared discussing only the first option in detail and not revealing alternatives if the client seemed to believe the first option presented to them was feasible. A couple of participants deviated from all other participants by describing tailoring their presentation of options to a client by basing the options they presented on the history they gathered prior to conversations around decision-making. Another few participants described offering a recommendation and only providing alternatives once their recommendation was rejected. Finally, all participants described situations in which presentations of options were limited by the patient’s medical status. Each of these approaches is detailed as subthemes below.

**2.1 Subtheme: Present all available options upfront alongside risks, benefits, and other details**: Most participants described the importance of outlining upfront all options (e.g., *“lay*[ing] *out option A, B, or C” (P1)*), in some detail including common side effects, risks, benefits, and medical implications, while *“checking in and see*[ing] *how they’re feeling about each of the options” (P5).* These participants describe this in contrast to waiting for a client to select or reject an option before presenting the next. These upfront options were described as being presented to clients in a stepwise fashion. Participants described this stepwise fashion as incremental or cumulative options, least involved to most involved, or best to worst, as appropriate. These participants shared that this approach enables them to incorporate their client’s perspective. As one participant described: *“We really make a point of always giving people like multiple treatment options and estimates and kind of explaining the difference between them. And then I always tell owners that I look at it as it’s my job to give them all the options and their job to decide which one is right for them” (P4).*

After stating that a range of options are generally offered, participants were probed to share specifics of how they present options. In situations when clients were eager to move forward with the first option presented, of participants who stated presenting a range of options, two-thirds indicated that they still shared the remaining options, in detail or briefly with that client. One participant summarized, *“I … still feel the necessity to point out their options… I wouldn’t want to seem to be changing their mind, but I’d want them to know” (P15).* The other one-third of participants described moving forward with the first option presented only, if the client was eager. Otherwise, if the client did not show overwhelming interest in the first option, these participants also described proceeding with discussing other options and gaining client perspectives on each one. This approach was shared as preventing clients from feeling pressured into an option implausible for them to adhere.

**2.2 Subtheme: Make a recommendation and discuss other options, if needed**: In contrast to the intention of making clients aware of all available options upfront, as described above, a few participants expressed that they begin by discussing only their primary recommendation (i.e., option). After this first recommendation has been responded to, by the client, and if accepted, no other options are presented. If a client indicates the recommendation is out of reach, these participants described then proceeding to outline or present alternative care options. All these participants, at multiple points during their interviews, expressed that they *“always” (P2)* offer a range of options, going on to correct themselves by indicating they discuss alternative options only when an initially proposed plan is met with resistance from the client. One participant stipulated that this resistance ranges from client’s verbal disagreement or the veterinarian noticing non-verbal feedback, including client pauses (i.e., non-immediate agreement). A participant who shared use of this technique described this general approach as, *“It would be my version of gold standard that I always would like to present first…*[then] *we get feedback from them* [the client] *as to whether that’s feasible or not. So, it’s a discussion” (P14).*

A couple of participants shared that this suggestion of a recommendation and lack of a discussion around options was done only in some cases, where clients were adamant that they did not want to discuss alternatives. One participant clarified that, for them, a singular option was offered in a few instances with long-term clients, who had previously almost always consented to care. Furthermore, these participants described using this approach of focusing on a recommendation to save time and prevent overwhelming or confusing the client. As one participant articulated: *“If they* [the clients] *are willing to spend money, then you* [the veterinarian] *don’t always have to spend as much time explaining the value of what you’re doing and giving all the options and like finessing your final plan” (P11).* All of these participants shared that they ultimately end up discussing options, because most clients share barriers to their initial recommendation.

**2.3 Subtheme: Contextualizing options prior to presenting them:** In presenting a range of options, prior to presenting any options to a client, a couple of participants described gathering information from the client to tailor the range of options presented and to avoid assumed or previously discussed client barriers. Specifically, these participants shared that their approach to providing options was to utilize information collected during the history-gathering portion of the appointment, in order to focus on presenting clients with multiple options tailored to the patient and client’s situation. One participant described this method, sharing that *“I will be sitting there, getting a fairly good history and then doing a physical and by that time—most times—you have an idea of which way you want to go… I say, ‘this is what you could do.’ I usually give a few options. I don’t get upset or try and force them to do anything, even though I know they could afford it or something like that... But I will sometimes speak up, and say, if I give them three options, ‘this is one I would not want* [you] *to do.’” (P3)*

Some other participants stated that they avoid the technique of contextualizing options in their own mind prior to presenting options. These participants who preferred not to tailor options prior to a decision-making conversation described the approach as part of mitigating their own perception bias of what they perceive their client could afford or would prefer.

**2.4 Subtheme: Presenting options is not always possible**: All participants identified that there are situations where they are limited by the patient’s case in terms of available options. Participants indicated these situations were often based on case severity. In both mild or severe cases, participants shared that there were only two clear options: basic treatment or monitoring, and euthanasia or surrender. A few participants summarized this concept as cases in which *“that middle one* [option] *kind of goes away” (P6).* A few participants shared that options may not be offered for diagnostics in cases where inexpensive and straightforward treatment options are available, with one of the participating veterinarians providing an example of a *“puppy with mild diarrhea, that owner gave some human food to yesterday” (P5).* Other participants provided a similar example and added that an additional option provided could be to monitor the patient at home. As such, participants often identified that even in discussion of situations characterized as not needing options, more than one option could still be found and presented as needed. Almost half of participants described it in the context of these basic cases, where the options become providing a basic treatment or monitoring. Most participants shared that euthanasia may be the only option in severe cases, with direct and immediate suffering involving intensive injuries, since “*in some situations it becomes a question of quality of life” (P2).* In these instances, surrender was noted by a couple of participants in conjunction with euthanasia to arrive at the same result of easing severe pain through possible end of life care or intensive measures. A third of participants noted an additional case in which options were limited: abuse. In this case, reporting to relevant agencies or providing care were the only options these participants identified.

#### 3. Theme: Veterinarians and team members face challenges when presenting options to clients.

Participants described multiple challenges to presenting options to clients, including time constraints, client “non-cooperation”, and personal bias held by veterinarians. Veterinarians also outlined the *“emotional toll” (P4)* they and their team members take on when client barriers prevent the pursuit of care the veterinary team considers acceptable.

**3.1 Subtheme: Appointment duration constrains time to discuss options**: Two-thirds of participating veterinarians identified time as a challenge for presenting options. One participant summarized this general perspective, stating: *“It does take a lot of time to have that informed consent and that is the part I do find challenging is finding the time to go over everything*” *(P5).* A handful of participants described needing to extend the duration of their appointments over their time in practice to accommodate presenting options to ensure they are meeting the requirements of providing informed consent. Others shared that they delegate communication of additional details regarding consent. For example, a couple participants described delegating conversations of cost to support staff.

**3.2 Subtheme: Veterinary client** *“non-cooperation” (P1)* **when discussing options**: Over two-thirds of participants noted *“owner non-cooperation” (P1)* as a challenge when offering options, which they defined collectively as clients being resistant to hearing options. Participants described non-cooperative clients who immediately decline each option, before opting to take their pet home without care, and some clients who do so in a confrontational manner. As a solution to this resistance, participants described being emotionally prepared for clients who enter the practice frustrated. When engaging clients who participants described were not *“willing to listen” (P7)* and were inclined to reject care, two-thirds described attempting to discover their client’s bias or limitations regarding care, through asking questions. One of these veterinarians specified that if no information is shared by clients in response to questioning, that they (the participant) do not probe further or provide additional information. Engaging in discussion to identify a client’s bias or limitations was considered helpful by a handful of participants when offering options, as it assisted in addressing client hesitations around care options or preferences, with participants sharing examples of discussions around raw-food diets or with clients having consulted *“Dr. Google” (P9).* One participant described this approach as *“talk*[ing] *to the client on a person-to-person basis and saying, ‘What are you thinking right now?’ We* [the veterinarian and the client] *have a real conversation… You’re listening to what the client’s actually saying to you and then incorporating that into what’s best for the pet” (P7).* Another participant added that *“addressing the* [client’s] *stress is probably the most important thing to do in an appointment just because if that stress is not dealt with then it’s very hard for them to feel understood and then feel open to making a decision of how to try to fix things* [with the pet]*” (P13).*

**3.3 Subtheme: Veterinarians’** *“perception bias” (P6)* **influences options offered**: Over half of participants acknowledged their own biases as a challenge to presenting a range of options, of which they tried to maintain an awareness. As one participant described, *“where, if I look at one person and they look less wealthy, I may not suggest the Gold Standard as opposed to the person with the iPhone and the Mercedes-Benz” (P6).* Mitigating bias was mentioned as being necessary by over two-thirds of participants, in order to offer consistent care to all clients. Over a third of participants, when reflecting on the topic of mitigating their own personal biases in relation to discussing options with clients, shared their belief that recognizing bias is especially important when practicing in rural or demographically diverse areas. These participants compared their experiences discussing options with clients to those of colleagues working in practices where clients were generally financially more well off or motivated by other factors and were more likely to pursue recommended care.

Alongside participating veterinarians’ concern regarding their own bias, a few participants shared concerns with other practitioners who do not provide options. One participant shared: *“just seeing my own colleagues, I’ve had a range. So, we have like a locum right now that it’s gold standard or it’s nothing… And they can’t see beyond that. But I think after a while they’ll come to realize that not everybody can do that” (P7).* They described experiences within their practices of having clients who were not presented options, expressing that the client felt pressured into a care plan that was not feasible for them to maintain. These participants cautiously speculated reasons why other practitioners may be hesitant regarding discussing options, listing potential causes such as: practitioners’ resistance to change, time constraints, pressures from practices to maintain appointment times and revenue, a lack of comfort in presenting options due to this skill not being taught at veterinary colleges, and believing a range of options is not required due to affluent financial status of the area being served.

**3.4 Subtheme: The** *“emotional toll” (P4)* **on the team when prevented from providing care to patients**: When asked about the impact from clients who decide not to pursue care, two-thirds of veterinarians expressed the frustration they and their staff have experienced when not being able to provide what they believe to be an *“acceptable quality of care” (P2)*. A couple of participants also explained that discussing options to find a way for the animal to have a chance to heal or reduce suffering has reduced their *“emotional toll” (P4)* and their own risk of burnout. Additional participants shared how their experience and time in practice assisted them in mitigating frustrations by emotionally preparing them for difficulties, as compared to the limited experiences of newer team members.

#### 4. Theme: A shift away from a singular “Gold Standard” option.

All interviews involved a discussion of the GS, with over a third of participants describing a cultural shift away from providing a singular GS option, and moving towards the provision of multiple options.

**4.1 Subtheme: A cultural shift toward providing options**: Over a third of participants reflected on their experience of veterinary practice changing since they initially attended veterinary college, and described an experienced shift from a time when practitioners were *“guessing what* [pets and] *people need” (P8)* to discussing options. Half of those participants described that this change was being driven by current pet owners’ expectations, compared to when they were first in practice. One participant characterized this culture shift, sharing that *“at one time you said something, and they* [the client] *said, ‘OK, this is what we have to do.’ Now,* [clients] *want a reason why we have to do it” (P9).* These participants described comparing options as a means to meet the client expectation of being provided that ‘why’. Participants shared that this culture shift toward options may be connected to financial considerations, with clients portrayed by participants as being more mindful of budgeting due to rising costs of care, and thereby, expecting options that account for differing budgets. A handful of participants, all more recent graduates, suggested veterinary education may be contributing to this culture shift as they have always offered a *“range of options” (P8),* due to the education they had received. As one participant reflected on their veterinary education: *“I think our university put a lot* [of emphasis] *in terms of client communication and decision-making and making sure that it’s…informed consent. So, I think a lot of us make sure that decisions that are made by the client are informed, and the only way of doing that is taking the time to explain to them all their options and why those options are important” (P13).*

**4.2 Subtheme: Veterinary practitioners are redefining the Gold Standard**: Before being asked, the term ‘Gold Standard’ (GS) arose in discussion, organically, with over a third of participants including the term within their description of how they make decisions with clients. All participants were further probed to share their thoughts on the term, where it was defined broadly by participants as often the most expensive; *“best of the best care” (P6)*; determined by specialists, textbooks, or educational facilities*.* Most participants shared that it was in veterinary college when they were introduced to the term, with the remaining participants expressing that the term was introduced to them through continuing education attended, where it is also reinforced and further defined. Most participants used the term interchangeably with referrals to specialty hospitals, equating the ‘best care’ with often-expensive options that are unavailable through general companion-animal practices. Participant agreement varied on the GS being *“best for the animal” (P9)*, when discussing their thoughts on the GS. Most participants described a lack of practicality or necessity for the GS; whereas a small number of others viewed the GS as a useful option. A handful of participants emphasized their effort to keep up on the current GS, using literature and other resources to provide GS care. The majority of participants characterized learning about a single GS as a disadvantage, because it encouraged them not to account for client or patient barriers. Participants provided examples, such as patient behaviour, the human–animal bond, client financial constraints, or availability of diagnostics and treatments across veterinary clinics. As such, almost half of participants re-defined the GS to more-so reflect a current *“Gold-Centred” (P8)* approach to practice, focused on what is best for the patient. These same participants viewed the notion of the GS learned in veterinary college to be the *“Ivory Tower” (P11, P12)* GS or the *“*[Veterinary College Name] *standard” (P3, P11)*. A more experienced participating veterinarian shared their view on why practitioners are redefining the GS, explaining *“When we’re in school, it’s like ‘we’re to do ultrasound, X-rays, bone marrow biopsies and all these things.’ And these are very fearful situations for these animals and it’s a lot that they go through. So,* [in practice] *we like to make sure: is it necessary? So, I think that* [definition of the GS] *shifts, but initially your gold standard is based only on the medicine, it’s not really based on the client experience or the animals” (P12).* Most participants shared that experience in practice allowed them to gain comfort in discussing options with clients and deviating from the GS. Over a third of participants described the lack of instruction on presenting options as an area for improvement by veterinary colleges, to ensure students receive education on practical care options relevant to general practice for when the GS may not be accessible for clients. These participants proposed having students practice discussing and prioritizing options as a way to benefit students’ learning about *“all* [options] *that would have been optimal standards for your* [client and patient’s] *situation” (P9)*.

Most participants explained that, as they gained experience practicing veterinary medicine, their ability to adjust a treatment plan improved, including addressing the client’s budget and expectations, and being aware of available options. Determining what options were presented to clients for diagnostics and treatment was a matter of veterinarian experience, as veterinarians learn of more available options with increased time in practice and their comfort increases when discussing certain options (i.e., learned techniques). As stated by one participant who held this view, *“that’s* [determining options] *just experience and knowledge that if we’re* [the veterinarian] *dealing with a condition, I know we could do option A, B, or C” (P1).* Veterinarians described presenting clients with options, alongside a GS option, that they themselves considered acceptable (i.e., being able to bring the animal to better health). Furthermore, a few participants added that they may present an option they consider to be of lower acceptability, as a comparator in discussing the value of care recommendations.

**4.3 Subtheme: It is still important to offer the Gold Standard**: In almost all interviews, the GS or re-defined GS was brought up as being important to include as one of the multiple options presented to clients. Over two-thirds of participants described making the GS their top recommendation due to their desire to maintain consistency of care, mitigate personal perception bias, and ensure informed-client consent by making clients aware of the ideal, recommended care that is considered best for the animal. As well, the majority of participants described including the GS as one option to prevent clients from registering a complaint based on not being offered the GS. Furthermore, in discussing the GS, two-thirds of participants described their fear of missing an essential part of diagnosis or treatment and being at fault for inadequate care, as a result of not delivering the GS. This general fear of not offering the GS alongside other options was summarized by a participant, as follows: *“I think the fear is that I’m not being a good vet and fear that if you haven’t verbally mentioned all the options, then you could have backlash… So, it’s the fear* [of] *judgement from other clinicians, reading your record and being like, ‘Oh my God, why didn’t they do this battery of tests? And then fear that the client or college will see it as a failure on the veterinarian’s part to not offer all of the feasible options” (P11).* Nearly half of these participants described this concern as possibly being less notable for them compared to other practitioners, because they felt confident in their practice, as experienced practitioners, and in their documentation*.*

#### 5. Theme: Participants’ thoughts on the American Association of Veterinary Medical Colleges’ definition of Spectrum of Care.

When participants were asked directly, as part of the interview, about the terms SpoC, IC, and CC, most participants stated that they had not heard of any of those terms. The few participants who had heard of IC or CC, shared that they did not know the definition of those terms. Over a third of participants recalled hearing the term ‘Spectrum of Care’, and of these, a couple of participants produced a description of SpoC in line with the definition provided by the American Association of Veterinary Medical Colleges (AAVMC) prior to being given the AAVMC definition within the interview (Table 1). Before the term was presented to participants, ideas associated with SpoC were mentioned throughout every interview, including in relation to how most participants described their decision-making process for cases in which clients mention barriers or additional considerations.

When presented with the AAVMC definition of ‘Spectrum of Care’ (Table 1), all participants stated that SpoC captured their current practices and their expectations of other veterinarians’ practices. One participant responding to the AAVMC definition, captured the response of many participants, stating: *“it’s more realistic and it allows space for the client’s own financial or emotional or personal/spiritual beliefs to come into play. There are just genuinely some people who, even if they have all the money in the world, they wouldn’t do Gold Standard. Because for whatever reason for them or their pet or their beliefs, it’s just not something they would do. So, this opens the doorway for the client to feel supported even though they don’t just go for the gold” (P11).* One participant added that they believed there is a limit, indicating that providing space for clients to deviate from recommendations and putting the onus on the veterinarian to address client barriers is not always ideal.

## Discussion

Results of this study provide an understanding of participating veterinarians’ perceptions of veterinarian-client decision-making in the context of providing access to veterinary care. Many of the participating veterinarians discussed offering options in varying ways and described utilizing conversation with clients around options to contextualize care and overcome multiple barriers to accessing veterinary care. By presenting options, a number of participants believed it allowed them to consider unique factors specific to the individual client, patient, and their situation that necessitated care plans to be contextualized. Spectrum of Care is a defined approach to practicing veterinary medicine that aims to provide a continuum of acceptable care options that balance client circumstances and expectations with evidence-based medical options [13,14]. The findings of the present study directly relate to the idea of practicing Spectrum of Care and suggest that, while practicing veterinarians may not be broadly aware of the term SpoC, participants generally agreed with the definition, recognized its importance, and believed it to be consistent with their current practice of discussing options. In sharing their own approaches to decision-making, every participant described aspects of communicating SpoC. This indicates a potential growing recognition, occurring organically, within companion-animal practices, for discussing options and practicing SpoC. Participants of the present study described either that the idea of providing more than one option arose for them from experiences in practice where they developed an understanding of the need to contextualize care, or for other participants, often newer graduates, from their veterinary college education. While recommendations have been made that veterinarians provide options [19–21] and move away from presenting only a singular GS option [22,23], findings of the present study suggest there are already veterinarians moving toward practicing or aiming to practice SpoC, without formal awareness of the concept.

The present study also highlighted participants’ views on the function of information gathering as part of decision-making and its role in providing accessible care. Incorporating client knowledge of animal-specific barriers (i.e., their pet’s behaviour and preferences) in these decision-making discussions allows for a tailored care plan to be created and adhered to, thereby, improving the patient’s access to care, as was emphasized by participants in the present study. Several participants identified understanding a client’s perspective in providing individualized care as essential, as it leverages their client’s expertise of their pet in order for feasible care plans to be tailored to the client, the patient, and their situation. One study examining clients’ responses to nutrition recommendations during video-recorded consultations found participating clients involved in the interactions often demonstrated resistance to the veterinarian’s nutrition recommendation [24]. When the basis for clients’ resistance was further explored, many of the issues were identified to be things that could have been learnt and incorporated into the recommendation with a more comprehensive history. As described by some participants in the present study, gathering information from the client is a crucial first step in individualizing care and integrating a client’s perspective. This includes client knowledge of the patient, which was noted by over half of participants in the present study. Previous research on medication compliance found that noncompliance for administration was recorded in nearly 40% of cat owners, with the most common reason for challenges in administration being a resistant pet [25]. As such, patient behaviour, and more specifically a client’s perception of their pet’s behaviour, is an important factor to be considered in veterinarian-client decision-making and in creating a care plan that is tailored to an individual client, patient, and their situation. Furthermore, a veterinarian’s history taking plays an important role in veterinarian-client decision making, and in turn providing access to care by allowing the practitioner to integrate client, patient, and unique situational factors into overcoming barriers to creating feasible and accessible care plans for the client.

Most participants in the present study described presenting options to clients, alongside presenting each option’s risks and benefits. This description reflects participants’ emphasis on ‘educating [clients] about options’, which is an element of the 3-talk model of SDM described in human medicine [26]. In an assessment of 717 veterinarian-client-patient interactions, using the Option5 tool adapted from human medicine for evaluating SDM, option talk was the aspect of SDM most utilized in these interactions compared to other components of SDM [27]. Yet, overall SDM was found to be uncommonly implemented [27]. The other two elements in the 3-talk model include “team-talk”, where it is clear a decision is to be made between the client and veterinary professional, and “decision-talk”, where a choice is contextualized to client preferences and a preference-sensitive decision may be made [26]. Communication consistent with a SDM approach aligns with many of the elements associated with obtaining informed-client consent (ICC) [27]. Notably, a number of participating veterinarians recognized the role of discussing options in achieving ICC. The discussion of risks and benefits of care alternatives, alongside costs are also important components for obtaining ICC [28]. Through further uptake of the elements of SDM to discuss options and integrate client preferences, veterinary professionals can be supported in achieving ICC.

In contrast to presenting options, some participants described starting the decision-making conversation by making a single recommendation to the client. As such, it should be noted that providing a singular option, GS or otherwise, does not align with criteria for obtaining ICC [28]. Interestingly, the majority of participants in the present study described including the GS as an option to prevent complaints being registered with a regulatory body. Importantly, regulatory bodies have begun to acknowledge support of veterinarians in presenting options and in taking a SpoC approach [29,30]. In their support they acknowledge that multiple options for care often exist and that these should be communicated while considering the patient’s case, and client and clinic capacities [29,30]. Furthermore, it has been acknowledged within veterinary medicine that cases can vary between minor and serious, acute and chronic, and therefore there is no singular best approach towards assisting all clients with decisions across all situations and all circumstances [31]. As such, improving access to care is more likely to be achieved through the presentation of options, which includes educating clients about each option, integrating client preferences, addressing barriers, and obtaining informed-client consent.

To this end, participants described utilizing the decision-making conversation involving options to identify barriers (e.g., affordability, client capacity to provide care [i.e., their “swirl”], knowledge-based barriers [i.e., those related to communication], and animal-specific barriers). Many of these barriers identified by participants (e.g., cost of care, transportation issues, client capacity for care, understanding importance of care, and patient-specific considerations) have been recognized barriers to AVC [3,4,6,13]. Within the present study, discussing options and identifying client barriers were described as beneficial to providing AVC options that were considered acceptable by a participant and their staff. An opportunity exists to make further progress towards broadening access to veterinary care by presenting options effectively, incorporating client preferences, and addressing barriers in discussion of options explicitly. SDM is one communication approach to achieve each of these goals and is thereby likely to lead to greater AVC [32]. Practicing veterinarians should be encouraged to consider the use of SDM with a client regardless of a client’s past decisions or capacity to pursue care. Available resources [33] and continuing education may be valuable to support veterinarians in continuing to adapt to providing SpoC, including the use of SDM to discuss options and address barriers with clients.

Participating veterinarians identified challenges to presenting options to clients including time constraints, un-cooperative clients, and the emotional toll on the veterinary team when a client elects not to provide care to a patient. Participants recognized the impact of appointment duration and time within the veterinarian-client-patient interaction on communicating options and obtaining ICC. Time has been an identified challenge for SDM, as one study found that increased appointment duration was positively associated with elements of SDM utilized within veterinarian-client interactions [27]. For clients who may not appreciate hearing all options or hold strong preferences regarding certain alternatives, approaches to discussing options may be individualized to these preferences. Preferences may also shift for each client, depending on their own capacity or the patient’s case [34]. Two-thirds of participants shared that they integrate these preferences or explore options more with these clients through asking questions to uncover potential client concerns or limitations. Participants acknowledged that, given the emotional distress experienced by them and their team when not able to provide care to a patient, by offering options instead of focusing on one solution, clients have a greater likelihood of pursuing an accepted level of care.

While solutions to challenges were not discussed in-depth by participants of the present study, participants acknowledge that if they are able to educate clients on options, some of these challenges can be mitigated. Offering options has been proposed to potentially decrease moral distress (i.e., the emotional and psychological suffering veterinary team members experience when they are unable to provide care in line with their personal ethics) and increase veterinarian satisfaction, by enabling contextualized, appropriate care to be provided to patients [35]. A mixed-methods study examining moral distress in North American veterinarians found 70% of respondents reported that barriers to providing appropriate care caused them, or members of their staff, moderate to severe moral distress [36]. Educating veterinary professionals on SpoC, including new graduates, other practitioners, and staff members, may help to mitigate moral distress for all staff. This was highlighted by a couple of participants in the present study who shared that they felt offering options reduced their risk of burnout, as they felt they were still able to help the animal. Embracing this new culture surrounding the practice of SpoC may alleviate some of the challenges identified by participants and improve the wellbeing of veterinary teams.

Semi-structured interviews were employed to understand participating veterinarians’ in-depth perspectives on the topics of decision-making and accessing veterinary care. These in-depth hypothesis-generating insights offer direction for future research. As with all qualitative research, given the hypothesis-generating nature of the current work, these results are not intended to be generalizable. Although generalization of results is not possible, nor intended, the random sample of veterinarians contacted was used to facilitate the collection of perspectives from a diverse sample (i.e., years in practice and experience, demographics of clientele served). As data saturation was reached, the rigor of the findings assessed is supported for the population of veterinarians recruited to the present study. Given the focus of the present study on veterinarians’ perspectives, future research is also needed to qualitatively explore veterinary clients’ perspectives on these topics.

Results of the present study emphasize participating veterinarians’ view on the important role decision-making plays in enhancing AVC. Veterinarians participating in the present study identified options as an approach for overcoming multiple barriers to accessing veterinary care for clients and patients. Participants suggest that the practice of presenting options is potentially becoming more common, although how options are being presented by veterinarians varies. Findings of the present study align with the process of SDM, which involves veterinarians engaging with clients regarding care options, understanding individual client preferences and capacities, utilizing client’s knowledge of the patient and patient’s situation in order to provide feasible, accessible care plans tailored to an individual client and their animal. Best practices should be explored for providing and communicating evidence-based, contextualized access to veterinary care options.

## Supporting information

S1 File Veterinarian demographic information questionnaire. (PDF)
