# Supplementary material for: Exploring veterinarians’ perceptions of decision-making with clients in the context of providing access to veterinary care
Source: PLoS One. 2026 Feb 12;21(2):e0342564. doi: 10.1371/journal.pone.0342564 (PMC12900299; doi:10.1371/journal.pone.0342564)
Supplement: S1 File — (PDF) [file pone.0342564.s001.pdf]

Veterinarian Demographic Information Questionnaire

I identify my gender as:

- Man (1)
- Non-binary (2)
- Woman (3)
- Prefer to self-describe, please specify (4)

  ____

- Prefer not to say (5)

What is your current employment status?

- Employed full time (40 or more hours per week) (1)
- Employed part time (up to 39 hours per week) (2)

What year did you graduate from veterinary college?

____

What is your role at your practice?

- Owner (1)
- Associate (2)
- Locum (3)
- Prefer to self-describe, please specify (4)

  ____

Do you work strictly as a small animal practitioner?

- Yes (1)
- No (2)

Display This Question:

If Do you work strictly as a small animal practitioner? = No

If no, what percentage of your time is spent practicing small animal?

- Less than 25% (1)
- 25-50% (2)
- 50-75% (3)
- Over 75% (4)

How many other veterinarians work at your practice?

- Full time (1) ____
- Part time (2) ____

How long are your appointments scheduled for, on average?

____

What is the average cost of an examination at your practice? ($)

____

Have you had any communications training?

- Yes (1)
- No (2)

Display This Question:

If Have you had any communications training? = Yes

Please describe your communications training:

____
